# Incident experience predicts freezing-like responses in firefighters

**DOI:** 10.1371/journal.pone.0186648

**Published:** 2017-10-18

**Authors:** Verena Ly, Linsey Roijendijk, Hans Hazebroek, Clemon Tonnaer, Muriel A. Hagenaars

**Affiliations:** 1 Department of Clinical Psychology, Leiden University, Leiden, The Netherlands; 2 Leiden Institute for Brain and Cognition, Leiden University, Leiden, The Netherlands; 3 Behavioural Science Institute, Radboud University, Nijmegen, The Netherlands; 4 TNO Human Factors, Soesterberg, The Netherlands; 5 Fire Service Academy, Instituut Fysieke Veiligheid (IFV, Institute for Safety), Arnhem, The Netherlands; 6 Department of Clinical Psychology, Experimental Psychopathology group, Utrecht University, Utrecht, The Netherlands; Swansea University, UNITED KINGDOM

## Abstract

Freezing is a defensive response to acute stress that is associated with coping and alterations in attentional processing. However, it remains unclear whether individuals in high risk professions, who are skilled at making rapid decisions in emergency situations, show altered threat-induced freezing. Here we investigated the effect of incident experience in a high risk profession on freezing. Additionally, we explored whether any effect of incident experience on freezing would be different for profession-related and -unrelated threat. Forty experienced and inexperienced firefighters were presented neutral, pleasant, related-unpleasant, and unrelated-unpleasant pictures in a passive viewing task. Postural sway and heart rate were assessed to determine freezing. Both postural and heart rate data evidenced reduced freezing upon unpleasant pictures in the experienced versus the inexperienced group. Relatedness of the unpleasant pictures did not modulate these effects. These findings indicate that higher incident experience relates to decreased threat-induced freezing, at least in a passive task context. This might suggest that primary defense responses are malleable through experience. Finally, these findings demonstrate the potential of using animal to human translational approaches to investigate defensive behaviors in relation to incident experience in high risk professions and stimulate future research on the role of freezing in resilience and coping.

## Introduction

Freezing is a major defensive response to threat in animals, which is characterized by heart rate deceleration (bradycardia) and immobility [1–5]. In general, freezing is considered adaptive as it optimizes attentional processes and prepares the organism to actively cope with acute stress [5–7]. Accumulative evidence suggests that freezing, as measured via reductions in postural sway and heart rate, can be induced in human subjects in a laboratory setting using aversive stimuli in a passive viewing task [8–13].These findings are robust; passively viewing aversive stimuli consistently elicits a decrease in heart rate and/or postural sway relative to neutral or pleasant stimuli. Additionally, a recent study has shown that bradycardia during passively viewing aversive pictures was associated with activation of the human periaquaductal grey and the amygdala, which overlaps with the neurocircuitry involved in rodent freeze behavior [14]. Altogether, these findings suggest that threat-induced freezing is a phenomenon that is preserved in humans.

Recent human studies have also demonstrated the adaptive nature of freezing. For instance, freezing in terms of heart rate deceleration was associated with enhanced detection of coarse versus detailed visual information, suggesting that freezing is accompanied by perceptual changes aimed to promote threat-coping [15]. Additionally, recent evidence indicates that freezing reflects an active state rather than a passive state [16]. Specifically, it was found that freezing, as reflected by reductions in heart rate and postural sway, was more pronounced under threat when an opportunity to counter the threat was anticipated compared to a helpless condition without such opportunity. Thus, this finding suggests that freezing plays an important role in action preparation. In line with this, using mental imagery to prepare participants for subsequent negative stimuli also increased freezing upon these aversive pictures, possibly reflecting action preparation after mental imagery of the anticipated aversive pictures [17]. Although these findings indicate that threat elicits freezing, and that this response is important for active coping, the ability to (down)regulate this primary defense response might also be crucial for adaptive responding [18–19].

Despite the relevant link between freezing and threat-coping, it remains unknown how threat-induced freezing manifests itself in individuals engaged in high risk professions. These individuals are trained to deal with emergency situations, such as is the case for firefighters. More often than the general population, firefighters are exposed to life-threatening events, such as when risking their own life to enter a burning building and witnessing the suffering of others. At the same time, firefighters are required to actively cope and make optimal instant decisions under these extreme situations. Investigating the relationship between incident experience in firefighters and threat-induced freezing is important as it could advance our understanding in the role of freezing in performance in high risk professions. More generally, it could help us better understand the role of freezing in resilience and coping.

In the present study, we took a first step to investigate the relation between incident experience and threat-induced freezing in individuals in a high risk profession. To this end, we compared experienced versus inexperienced firefighters on a well-established paradigm to determine human freezing-like responses [8–9, 20]. Specifically, we employed a passive viewing task involving neutral, pleasant, related-unpleasant, and unrelated-unpleasant pictures. Posturography and heart rate recordings were assessed during this task to determine freezing responses. The passive context of this task allows us to optimally assess freezing as a spontaneous response to unpleasant pictures rather than freezing as a preparatory response for action as would be the case in a context where actions are required. Additionally, we included both profession-related and –unrelated stimuli to explore whether any effects of incident experience on freezing would be specific for profession-related (versus profession-unrelated) threat or whether incident experience would have a general effect on threat-induced freezing.

## Methods

### Participants

We recruited male firefighters from the Dutch fire brigade, who were working as a battalion chief or junior incident commander. The main role of these fire officers is to direct and coordinate fires, and other rescue and emergency activities located in a specific region. Thus, we chose this population as the target group for the current study given their experience with directing emergency activities, which requires rapid decision making in emergency situations. Twenty experienced and 20 inexperienced fire officers were preselected based on the number of big fires they have coordinated in their current rank (≥ 4 for the experienced firefighters, and ≤ 3 for the inexperienced firefighters). A big fire was defined as involving more than three fire engines. Thus, the initial total sample consisted of 40 fire officers. However, five fire officers who were tested had to be excluded because of technical errors (*n* = 3), or poor signal quality of the heart rate data (*n* = 2). Accordingly, data are reported from thirty-five firefighters consisting of experienced (*n* = 19) and inexperienced (*n* = 16) fire officers. For a description of the characteristics of the sample, see Table 1.

**Table 1:** Demographic data (Mean and SEM).

	Experienced (*n* = 19)	Inexperienced (*n* = 16)	χ^2^ or *F*	*p*-value
Big fires	8.89 (0.99)	1.06 (0.25)	35.00	<.001
Age (in years)	44.68 (1.82)	40.56 (1.44)	2.98	.093
STAI-Trait	30.16 (0.97)	32.00 (1.17)	1.50	.229
TIS-TI	10.58 (1.6)	9.88 (1.50)	0.07	.749
LOT-R	22.11 (0.63)	22.00 (0.51)	0.02	.899

Note. Big fire: involving more than 3 fire engines; STAI = State-Trait Anxiety Inventory; TIS-TI = Tonic Immobility; LOT-R: Life Orientation Test-Revised.

As firefighters are regularly screened for physical and mental health, we only included healthy firefighters. Fire officers with a bodyweight above 120 kilograms were excluded from participation due to the limits of the stabilometric platform for accurate measurements. Furthermore, we excluded fire officers who experienced a big fire in the three weeks before the assessment to avoid acute effects of an incident. All participants received information about the experiment and gave written informed consent. The study was approved by the Institutional Review Board: the Ethical Committee Behavioural Sciences (Ethische Commissie Gedragswetenschappen in Dutch).

### Apparatus and material

#### Equipment for postural sway and heart rate measurements

A valid and reliable way to assess freezing-like behavior is by means of a stabilometric force plate, which quantifies spontaneous fluctuations in postural sway during threat exposure [8–9, 21]. Participants performed a passive viewing task on a custom-made strain gauge force plate (dimensions: 500 mm x 500 mm; sample frequency: 200 Hz), which consisted of four sensors measuring forces in the vertical direction. The signals from these sensors were used to derive time series of the center of pressure (COP) in the anterior-posterior (AP) and the mediolateral (ML) direction. Python 2.7.8 was used for posturographic data recording. Simultaneously, heart rate data was acquired during the passive viewing task using an Arduino pulse sensor attached to the fingertip of the right index finger. The signal was registered at a sample frequency of 200 Hz using Python 2.7.8 and LabVIEW. The visual stimuli of the passive viewing task were presented 1 m in front of the participants at eye level on a 22-inch height adjustable screen using PsychoPy v1.81 software.

#### Passive viewing task

Participants were instructed to stand upright and remain stationary on the board, barefooted with their feet next to each other approximately 30 cm apart. We instructed them to equally distribute their body weight on both legs with their arms hanging relaxed beside their body. At the same time, they were asked to focus on the screen to watch a sequence of stimuli on the screen. Before the actual task, participants viewed a sequence of six trials in which letters instead of pictures were presented in order to get familiar with the task. The passive viewing task was presented subsequently, in which four blocks with 20 pictures of one type of stimulus category (neutral, pleasant, related-unpleasant, unrelated-unpleasant) were presented consecutively for three seconds without inter trial interval. The order of blocks and stimuli was counterbalanced. Blocks were separated by a black screen (five seconds) followed by a white fixation cross (two seconds).

As visual stimuli, four sets of pictures (neutral, pleasant, related-unpleasant, unrelated-unpleasant) were used from the International Affective Picture System (IAPS; Center for the Study of Emotion and Attention, 1999). Every set consisted of 20 pictures. The neutral pictures consisted of pictures of objects, such as clothespins and a mug. The pleasant pictures consisted of pictures of sports, such as waterskiing and skydiving. The related-unpleasant set included pictures that were related to the work of firefighters, such as burnt bodies and fires; whereas the unrelated-unpleasant pictures depicted threatening scenarios unrelated to the work of firefighters, such as weapons and snakes. The categorization of relatedness was confirmed in an independent sample of firefighters (*n* = 5 experienced; *n* = 5 inexperienced; coordinated more or less than 3 big fires respectively), that rated the related-unpleasant and unrelated-unpleasant stimuli on a scale ranging from 0 = *unrelated* to 10 = *related* to their profession as indicated by a main effect of relatedness (*F*_(1,9)_ = 20.0, *p* = .002, *η*^2^_p_ = .690; *M*_related_ = 4.86, *SEM*_related_ = 1.06; *M*_unrelated_ = 1.22, *SEM*_unrelated_ = 0.35).

#### Questionnaires

We included several self-report questionnaires to assess individual differences in trait anxiety, tonic immobility, and optimism, because these factors have been related to individual differences in automatic stress responses [22–26]. We included these measures to test whether any group differences in freezing would be due to differences on these measures rather than differences in incident experience. Trait anxiety was assessed using the Spielberger State-Trait Anxiety Inventory (STAI) [27]. The STAI demonstrated good reliability and validity [27]. Participants responded to 20 self-reported items on a four point scale to indicate how anxious they feel in general. The Tonic Immobility Scale (TIS) was used to retrospectively assess reactions during a previous, most frightening, event [28]. Ten self-report items were scored on a seven point scale. Factor analyses have demonstrated that the TIS comprises two subscales: Fear (TIS-Fear; three items) and Tonic Immobility (TIS-TI; seven items) [29]. In the current study, we focused on the TIS-TI only, as we have previously shown that this subscale is a more valid measure of threat-related responses [24].Optimism was assessed with the Life Orientation Test-Revised [25]. This is a brief questionnaire consisting of ten items. Participants had to indicate to what extent the items applied to them on a five point scale. Of the ten items, four items serve as fillers. The questionnaire demonstrates acceptable reliability [25].

Subjective immobility was assessed by asking the participants to rate the extent to which they felt paralyzed when watching the picture on a scale ranging from 0–10 (0 = *not paralyzed at all* to 10 = *extremely paralyzed*). Furthermore, participants rated all picture stimuli on a scale ranging from 0–10 in terms of valence and arousal (0 = *extremely unpleasant* to 10 = *extremely pleasant*; and 0 = *not arousing at all* to 10 = *extremely arousing* respectively).

### Procedure

Prior to testing, we attached the fingertip pulse sensor to assess heart rate throughout the experiment. Participants were then instructed to sit down in front of a computer screen to watch a three-minute neutral film depicting nature scenes, in order to normalize heart rate. Participants were then instructed to remove their shoes and take their position on the center of the stabilometric platform (with their feet 30 cm apart) to perform the passive viewing task. Before the start of the task, standardized instructions were displayed on the monitor. After the passive viewing task, participants completed the questionnaires and stimulus ratings.

### Data- and statistical analyses

The analyses for posturographic and heart rate data were conducted using MATLAB R2009b (The MathWorks, Natick, MA). Statistical analyses were performed using IBM SPSS Statistics 23 (IBM Corp., Armonk, NY).

#### Demographic data

We used analyses of variance (ANOVA) to test for any differences on the group characteristics.

#### Freezing

Following previous work, freezing was defined as a reduction in postural sway and heart rate [8–9, 21, 26]. Posturographic data were bandpass filtered using a 3rd order Butterworth filter with 0.01–10 Hz as a cutoff frequency. The standard deviation in anterior-posterior direction (SD-AP) was then determined for each stimulus, after which a mean SD-AP was calculated per stimulus category, with lower values representing increased freezing to the relevant stimulus-type. To calculate the heart rate per stimulus category for each participant, we analyzed the plethysmography data in the frequency domain using Welch’s periodogram method for each stimulus category condition separately [30]. The power spectral density distributions of the plethysmography data were calculated using a range from 0.7–2.4 Hz. Next, for each stimulus category, beats per minute (BPM) were calculated via the peak frequency multiplied by 60. Following previous work [21, 26], these mean SD-AP and BPM per stimulus category were used as variables in the following statistical analyses.

Two separate mixed design ANOVAs of postural sway (SD-AP) and heart rate (BPM) data were run to test the effects of stimulus category and incident experience on freezing. Stimulus category (neutral, pleasant, related-unpleasant, unrelated-unpleasant) was entered as a within-subject factor, group (experienced, inexperienced) as a between-subject factor, and SD-AP and BPM as dependent variables.

#### Subjective ratings

We ran three separate mixed design ANOVAs with stimulus category (neutral, pleasant, related-unpleasant, unrelated-unpleasant) as a within-subject factor, and group (experienced, inexperienced) as a between-subject factor to test the effects of stimulus category and incident experience on the subjective picture ratings in terms of immobility, valence, and arousal.

Importantly, threat-induced freezing was operationalized as freezing in response to unpleasant stimuli *relative* to neutral or pleasant stimuli in order to control for individual differences in postural sway and heart rate [8–9, 12, 21, 31–33]. Moreover, the pleasant stimulus category allows to control for arousal. Therefore, the crucial test is the group (experienced, inexperienced) x stimulus category interaction with pleasant versus (un)related pictures as the most relevant contrast for testing freezing behavior in experienced versus inexperienced firefighters. The crucial test for exploring the effects of profession-relatedness is the group (experienced, inexperienced) x stimulus category (related-unpleasant versus unrelated-unpleasant) interaction. For all analyses, significant interaction effects were followed up by simple (interaction) effects analyses. Multivariate test statistics were used in case of violation of sphericity. Alpha was set at .05.

## Results

### Demographic data

Table 1 presents the demographic data. Apart from incident experience (number of big fires), the groups did not differ on age and measures related to trait anxiety, tonic immobility, and optimism (all *F*s < 3.0).

### Freezing

Table 2 shows the mean data of the freezing measures in terms of postural sway (SD-AP) and heart rate (BPM). Both postural sway and heart rate data evidence a difference in freezing between the experienced and the inexperienced group as described below.

**Table 2:** Mean and standard error of mean per stimulus category of the freezing measures and the subjective ratings presented for the experienced (*n* = 19) and inexperienced (*n* = 16) group separately.

	Neutral	Pleasant	Unpleasant	
			Related	Unrelated
***Freezing***				
*Sway (mm)*				
Experienced	4.57 (0.38)	4.38 (0.34)	4.02 (0.27)	4.60 (0.42)
Inexperienced	4.47 (0.41)	4.24 (0.38)	4.03 (0.40)	3.81 (0.30)
*Heart rate (BPM)*				
Experienced	79.6 (3.2)	78.9 (3.2)	78.1 (3.2)	78.2 (3.2)
Inexperienced	82.3 (5.3)	82.7 (5.6)	79.0 (5.3)	79.7 (5.5)
***Subjective ratings***^a^				
*Immobility*				
Experienced	0.10 (0.07)	0.22 (0.12)	1.04 (0.28)	0.97 (0.36)
Inexperienced	0.24 (0.10)	0.93 (0.30)	2.53 (0.53)	2.00 (0.51)
*Valence*				
Experienced	5.48 (0.33)	6.74 (0.28)	2.84 (0.29)	3.12 (0.25)
Inexperienced	5.27 (0.25)	6.82 (0.16)	2.25 (0.23)	3.18 (0.25)
*Arousal*				
Experienced	2.42 (0.46)	4.74 (0.48)	4.83 (0.47)	4.37 (0.47)
Inexperienced	2.95 (0.47)	5.68 (0.38)	6.72 (0.26)	5.34 (0.43)

^a^Higher numbers indicate higher levels of immobility, pleasantness, and arousal

#### Postural sway

ANOVA of SD-AP revealed a main effect of stimulus category (*F*_(3,31)_ = 3.1, *p* = .043, *η*^2^_p_ = .229), but no main effect of group (*F*_(1,33)_ = 0.30, *p* = .586, *η*^2^_p_ = .009). Crucially, we found a significant group x stimulus category interaction effect (*F*_(3,31)_ = 3.1, *p* = .040, *η*^2^_p_ = .233). Follow-up analyses demonstrated a group x stimulus category interaction effect for the pleasant versus unrelated-unpleasant comparison (*F*_(1,33)_ = 8.6, *p* = .006, *η*^2^_p_ = .206), but not for the pleasant versus related-unpleasant comparison (*F*_(1,33)_ = 0.18, *p* = .677, *η*^2^_p_ = .005). The neutral versus (un)related comparison yielded a similar pattern (neutral versus unrelated-unpleasant: *F*_(1,33)_ = 3.9, *p* = .056, *η*^2^_p_ = .107; neutral versus related-unpleasant: *F*_(1,33)_ = 0.07, *p* = .794, *η*^2^_p_ = .002). Thus, experienced and inexperienced fire officers differed for the critical pleasant versus unrelated-unpleasant comparison, but not for the pleasant versus related-unpleasant comparison. The group x stimulus category interaction was not significant for related-unpleasant versus unrelated-unpleasant pictures (*F*_(1,33)_ = 3.1, *p* = .087, *η*^2^_p_ = .086), indicating that profession-relatedness of the pictures did not affect postural sway of experienced and inexperienced fire officers differently.

ANOVAs of SD-AP within each group separately suggested that the interaction effect was due to threat-induced reductions of postural sway in the inexperienced group (*F*_(3,13)_ = 5.5, *p* = .012, *η*^2^_p_ = .558), but not in the experienced group (*F*_(3,16)_ = 1.4, *p* = .273, *η*^2^_p_ = .211). Follow-up analyses in the inexperienced group demonstrated reductions in postural sway induced by the unrelated-unpleasant stimulus category (pleasant versus unrelated-unpleasant: *F*_(1,15)_ = 5.9, *p* = .028, *η*^2^_p_ = .283; and neutral versus unrelated-unpleasant: *F*_(1,15)_ = 7.1, *p* = .018, *η*^2^_p_ = .320), but not induced by the related-unpleasant stimulus category (*F*s < 2.0).

#### Heart rate

Similarly, an ANOVA of BPM revealed a main effect of stimulus category (*F*_(3,99)_ = 10.8, *p* < .001, *η*^2^_p_ = .247), but no main effect of group (*F*_(1,33)_ = 0.13, *p* = .718, *η*^2^_p_ = .004). Importantly, we also found a group x stimulus category interaction effect (*F*_(3,99)_ = 2.9, *p* = .040, *η*^2^_p_ = .080). Follow-up analyses demonstrated a significant group x stimulus category interaction for the pleasant versus related-unpleasant comparison (*F*_(1,33)_ = 6.6, *p* = .015, *η*^2^_p_ = .167), and a near-significant effect for the pleasant versus unrelated-unpleasant comparison (*F*_(1,33)_ = 3.8, *p* = .059, *η*^2^_p_ = .104). Neutral versus (un)related group x stimulus category interaction did not reach significance (neutral versus related-unpleasant: *F*_(1,33)_ = 3.0, *p* = .091, *η*^2^_p_ = .084; neutral versus unrelated-unpleasant: *F*_(1,33)_ = 1.7, *p* = .205, *η*^2^_p_ = .048). Thus, experienced and inexperienced fire officers differed for the critical pleasant versus related-unpleasant comparison. The group x stimulus category interaction was not significant for related-unpleasant versus unrelated-unpleasant pictures (*F*_(1,33)_ = 0.46, *p* = .503, *η*^2^_p_ = .014), indicating that, like in postural sway, profession-relatedness of the pictures did not affect heart rate of experienced and inexperienced fire officers differently.

ANOVAs of BPM within each group separately suggested that the interaction effect was due to the presence of threat-induced reductions of heart rate in the inexperienced group (*F*_(3,45)_ = 8.6, *p* < .001, *η*^2^_p_ = .365), but not in the experienced group (*F*_(3,54)_ = 2.4, *p* = .080, *η*^2^_p_ = .117). Follow-up analyses in the inexperienced group demonstrated heart rate reductions induced by both the related- and unrelated-unpleasant stimulus category for the critical comparison with pleasant pictures (pleasant versus related-unpleasant: *F*_(1,15)_ = 16.2, *p* = .001, *η*^2^_p_ = .519; pleasant versus unrelated-unpleasant: *F*_(1,15)_ = 7.0, *p* = .018, *η*^2^_p_ = .319), but also for the comparison with neutral pictures (neutral versus related-unpleasant: *F*_(1,15)_ = 22.4, *p* < .001, *η*^2^_p_ = .598; neutral versus unrelated-unpleasant: *F*_(1,15)_ = 9.7, *p* = .007, *η*^2^_p_ = .392).

**Fig 1:**
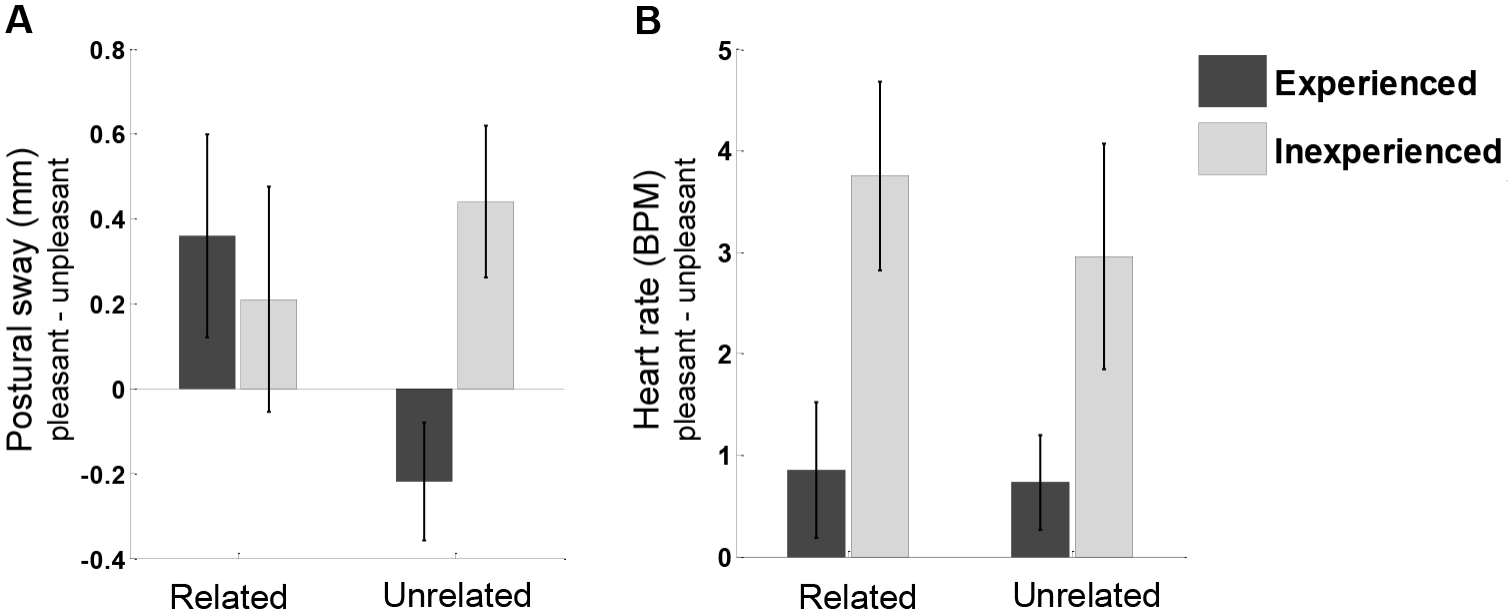
Freezing (pleasant versus related- and unrelated-unpleasant pictures) in terms of postural sway and heart rate for experienced and inexperienced firefighters. Positive bars reflect reduced sway or heart rate for (un)related-unpleasant pictures relative to pleasant pictures. (A) Inexperienced versus experienced firefighters show reduced postural sway (mm SD-AP) as induced by unrelated-unpleasant pictures. (B) Inexperienced versus experienced firefighters show reduced heart rate (BPM) as induced by related-unpleasant pictures. Error bars represent standard error of the mean.

Taken together, the postural sway and heart rate data evidence that threat-induced freezing was present in the inexperienced group but not in the experienced group. Specifically, the group difference in threat-induced freezing in terms of postural sway was mainly driven by the unrelated-unpleasant (versus pleasant/neutral) stimulus category, whereas the group difference in terms of heart rate was mainly driven by the related-unpleasant (versus pleasant) stimulus category. Crucially, the groups did not differ on the comparisons between related- versus unrelated-unpleasant pictures, indicating that relatedness of the unpleasant stimulus category did not modulate the effects of incident experience on threat-induced freezing. Fig 1 shows the group differences in freezing in terms of postural sway and heart rate for the most relevant contrasts (pleasant versus related- and unrelated-unpleasant pictures).

### Subjective ratings of stimuli

Table 2 shows the mean data of the subjective ratings with respect to immobility, valence and arousal.

#### Immobility

An ANOVA showed a significant main effect of stimulus category on immobility ratings (*F*_(3,31)_ = 10.7, *p* < .001, *η*^2^_p_ = .508). Follow-up comparisons demonstrated significant differences between all stimulus categories (neutral < pleasant < [unrelated-unpleasant, related-unpleasant]; all *F*s > 10.5, *p* < .003, *η*^2^_p_ > .241), except for the related-unpleasant versus unrelated-unpleasant comparison (*F*_(1,33)_ = 3.2, *p* = .082, *η*^2^_p_ = .089). A significant main effect of group indicated that inexperienced firefighters showed increased immobility ratings relative to the experienced firefighters (*F*_(1,33)_ = 5.6, *p* = .024, *η*^2^_p_ = .145). There was no significant group x stimulus category interaction (*F*_(3,31)_ = 2.7, *p* = .064, *η*^2^_p_ = .206).

#### Valence

Valence ratings differed across the stimulus categories (*F*_(3,31)_ = 65.2, *p* < .001, *η*^2^_p_ = .863). Post hoc comparisons demonstrated significant differences between all stimulus categories (pleasant > neutral > unrelated-unpleasant > related-unpleasant; all *F*s > 64.0, *p* < .001, *η*^2^_p_ > .660). There were no other main or interaction effects (all *F*s < 2.2).

#### Arousal

Also arousal ratings differed across the stimulus categories (*F*_(3,31)_ = 34.8, *p* < .001, *η*^2^_p_ = .771). Post hoc comparisons demonstrated significant differences between neutral versus all other three stimulus categories (neutral < [pleasant, unrelated-unpleasant, related-unpleasant]; all *F*s > 29.3, *p* < .001, *η*^2^_p_ > .471). No other comparisons reached significance (all *F*s < 2.8). A significant main effect of group indicated that the inexperienced firefighters rated all stimuli as more arousing than the experienced firefighters (*F*_(1,33)_ = 5.1, *p* = .031, *η*^2^_p_ = .134). There was no significant group x stimulus category interaction (*F*_(3,31)_ = 1.8, *p* = .164, *η*^2^_p_ = .150).

## Discussion

We found that inexperienced firefighters showed more freezing relative to experienced firefighters. Specifically, inexperienced firefighters demonstrated threat-induced freezing as reflected by reduced postural sway and heart rate in response to unpleasant pictures (relative to pleasant and neutral pictures), whereas the experienced firefighters did not show such response. Additionally, we did not observe any modulation by the profession-relatedness of the unpleasant pictures. These findings indicate that incident experience in firefighters was associated with reduced threat-induced freezing. Moreover, this reduction of freezing may not be restricted to profession-related threats, but possibly generalizes to other threats. Taken together, our findings may suggest that primary defense responses are malleable by training and experience.

Compared to inexperienced firefighters, our current experienced firefighters have received more education and training within their profession. Additionally, experienced firefighters have more experience with aversive events and experience in dealing with emergency situations relative to inexperienced firefighters. There are at least two mechanisms that could explain a reduction of threat-induced freezing through experience in firefighters.

First, experienced compared to inexperienced firefighters might show reduced threat-induced freezing due to an enhanced ability to control this primary defensive response. Although freezing to acute stress is adaptive, the ability to switch from immobility to active coping is important for adaptive responding [18–19]. This flexibility could be particularly important in high-risk professions, where optimal planning and decision making are necessary in emergency situations. Literature on controllability suggests that the ability to exert behavioral control over adverse events may enhance the ability to control this primary defensive response in future events [34–37]. In other words, experience of behavioral control has been shown to promote stress resilience that extends to other situations: even when one encounters an uncontrollable stressor later in time, the behavioral and neurochemical impact of the stressor is reduced via top-down inhibitory control [34, 36]. Such ability to control primary defensive responses could be accompanied by the recruitment of the instrumental system, which allow flexible behavior in a dynamic environment [18–19, 38]. By analogy, firefighters who are more experienced, have exercised behavioral control in stressful situations more frequently, and might therefore have more (perceived) capability of making rapid decisions under acute stress compared to inexperienced firefighters. Thus, one possibility is that experienced firefighters might have an enhanced ability to control primary defensive response, resulting in reduced threat-induced freezing. Future studies and more detailed time series analyses are needed to study effects of incident experience on inhibitory control on automatic defense behavior, as well as on the ability to switch between freezing and action.

Alternatively, experienced firefighters might show reduced threat-induced freezing due to faster processing of threatening stimuli. Rather than a passive state, freezing has been suggested to reflect an active state that promotes risk assessment in potentially threatening situations (5). Recent work has shown that under acute stress, freezing serves as a preparatory state for action [16,39]. Despite the use of a passive task context in the current study, in which active responses are not required, inexperienced firefighters might still demonstrate automatic preparatory responses upon the threatening stimuli. This is in line with the model of situational awareness, which suggests that automatically scanning for relevant clues in the environment plays an important role in gaining appropriate awareness of a situation in high-risk professions [40]. Thus, freezing in inexperienced firefighters could be crucial in real life situations as it facilitates adaptive responding to emergency situations. On the other hand, experienced firefighters might have developed an improved ability to process and integrate perceived information into decision making, which is reflected by reduced freezing in a passive task context that does not require action preparation. Future research is needed to disentangle the different hypotheses regarding the underlying processes of our observed effect of incident experience on threat-induced freezing. For instance, a worthwhile pursuit is to examine the effects of incident experience on freezing in passive versus active contexts.

Note that reduced threat-induced freezing was also found in patients with PTSD [26, 41] and borderline personality disorder [42] using passive viewing paradigms. In these groups, reduced freezing was assumed to reflect increased hyperarousal and vigilance and increased automatic fight-flight responses, which might attenuate adaptive freezing behavior. In other words, reduced threat-induced freezing might indicate impaired risk assessment and increased active responsivity to potential threatening stimuli, mediating cognitive distortions and exacerbated threat perception. However, it is unlikely that reduced freezing in our experienced firefighters is the result of a similar mechanism. First, we have tested healthy firefighters only, who are regularly screened for physical and mental health. Second, inexperienced firefighters reported higher arousal levels than experienced firefighters during picture viewing. Third, patients with threat-related psychopathology appear to suffer from impeded “downregulation”, as reflected by increased baseline or overall heart rate [42–44]. Our current heart rate data suggest that this was not the case for the experienced firefighters, as their overall heart rate was similar to (or if anything lower than) their inexperienced counterparts, rendering the inability to downregulate an unlikely explanation.

Our data did not provide any evidence for a modulatory effect of relatedness on freezing, although the profession-related pictures were rated as more related and also more aversive compared to the unrelated pictures across the whole sample. However, subjective ratings are more vulnerable to influences from awareness of experimenters’ demands and do not have to correspond with implicit and objective measures per se. Although it has been suggested that appraisal of relevance plays a crucial role in affective processing [45], the observed effect of incident experience on freezing might relate to alterations of other, perhaps more fundamental defense mechanisms. Therefore, rather than a specific group difference in freezing for profession-related (versus -unrelated) threat, the observed effect of incident experience was general to threat that is relevant to survival. In any case, we need to be careful with drawing conclusions based on a nonsignificant effect of relatedness, and further research on the relevance of relatedness in experience and freezing is warranted.

One could argue that our finding of increased freezing in the inexperienced versus experienced firefighters reflects an alteration in the degree of freezing in the inexperienced (rather than the experienced) group. However, findings with affective picture viewing typically found freezing in response to unpleasant pictures in healthy participants without high risk professions [8–9], suggesting that our observed effect of incident experience reflects alterations of freezing in the experienced firefighters. However, the question remains whether the inexperienced firefighters would resemble healthy participants without high-risk profession in terms of the degree of freezing. Future studies should include healthy participants without being involved in high risk professions as a control group to establish that incident experience indeed reduces freezing. Also, it is important to note that our sample consisted of battalion chiefs and incident commanders, who coordinate rescue activities and are responsible for their team. Although the experienced and inexperienced firefighters differed in their experience with the coordination of big fires, they have all built up substantial experience with firefighting-related tasks during their career. It is possible that experience with firefighting-related tasks from earlier ranks might also alter threat-induced freezing, contributing to a smaller difference between the groups in terms of threat-induced freezing in the current study. Future studies are needed to examine the effects of incident experience on spontaneous automatic defense responses in firefighters with lower ranks and thus less responsibility over the success of an operation.

The difference in freezing between experienced and inexperienced firefighters might be explained by an a priori difference between the groups in terms of trait and resilience rather than by incident experience or training, and that self-selection (through career switches) could have played a role in our findings. However, our questionnaire data do not suggest that this is the case as there were no differences between the experienced and inexperienced firefighters in terms of trait anxiety, tonic immobility, and optimism. To account for such interpretational limitations, we suggest that prospective studies are needed to elucidate more rigorously the effect of incident experience on defensive reactions to threat.

In sum, we have shown that higher incident experience in firefighters is associated with reduced threat-induced freezing. We did not find a modulatory effect of profession-related (versus –unrelated) threat on freezing. Although further research is needed to elucidate the mechanisms underlying our current findings, and replications are warranted given our small sample size, these findings help to bridge animal and human studies on defensive behaviors and indicate that primary defense responses are malleable by experience or training. Accordingly, the present findings represent an important step in inspiring future research into defensive reactions in high risk professions. The current findings demonstrate the potential of adopting animal to human translational approaches to assess defensive responses in relation to incident experience in high risk professions. Finally, these findings could stimulate the development of new (translational) procedures to test and train risk assessment abilities, which is particularly relevant in high risk professions.
